# Continuation rates and reasons for discontinuation of intra-uterine device in three provinces of Pakistan: results of a 24-month prospective client follow-up

**DOI:** 10.1186/s12961-015-0040-9

**Published:** 2015-11-25

**Authors:** Waqas Hameed, Syed Khurram Azmat, Muhammad Ishaque, Wajahat Hussain, Erik Munroe, Ghulam Mustafa, Omar Farooq Khan, Ghazunfer Abbas, Safdar Ali, Qaiser Jamshaid Asghar, Sajid Ali, Aftab Ahmed, Hasan Bin Hamza

**Affiliations:** Marie Stopes Society, Research, Monitoring and Evaluation Department, Technical Services, Karachi, Sindh Pakistan; Department of Urogynecology, University of Ghent, Ghent, Belgium; Research, Monitoring and Evaluation Department, Marie Stopes International, London, UK; Freelance Public Health Consultant, Adelaide, Australia

**Keywords:** Discontinuation, Family planning, Intra-uterine device, Social franchise

## Abstract

**Background:**

Long-acting reversible contraceptives, such as the intrauterine device (IUD), remain underutilised in Pakistan with high discontinuation rates. Based on a 24-month prospective client follow-up (nested within a larger quasi-experimental study), this paper presents the comparison of two intervention models, one using private mid-level providers branded as “Suraj” and the other using community midwives (CMWs) of Maternal Newborn and Child Health Programme, for method continuation among IUD users. Moreover, determinants of IUD continuation and the reasons for discontinuation, and switching behaviour were studied within each arm.

**Methods:**

A total of 1,163 IUD users, 824 from Suraj and 339 from the CMW model, were enrolled in this 24-month prospective client follow-up. Participants were followed-up by female community mobilisers physically every second month to ascertain continued IUD usage and to collect information on associated factors, switching behaviour, reasons for discontinuation, and pregnancy occurrence. The probabilities of IUD continuation and the risk factors for discontinuation were estimated by life table analysis and Cox proportional-hazard techniques, respectively.

**Results:**

The cumulative probabilities of IUD continuation at 24 months in Suraj and CMW models were 82% and 80%, respectively. The difference between the two intervention areas was not significant. The probability distributions of IUD continuation were also similar in both interventions (Log rank test: χ^2^ = 0.06, df = 1, *P* = 0.81; Breslow test: χ^2^ = 0.6, df = 1, *P* = 0.44). Health concerns (Suraj = 57.1%, CMW = 38.7%) and pregnancy desire (Suraj = 29.3%, CMW = 40.3%) were reported as the most prominent reasons for IUD discontinuation in both intervention arms. IUD discontinuation was significantly associated with place of residence in Suraj and with age (15–25 years) in the CMW model.

**Conclusion:**

CMWs and private providers are equally capable of providing quality IUD services and ensuring higher method continuation. Pakistan’s National Maternal Newborn and Child Health programme should consider training CMWs and providing IUDs through them. Moreover, private sector mid-level providers could be engaged in promoting the use of IUDs.

## Background

Long-acting reversible contraceptives (LARC), such as the intrauterine device (IUD), remain underutilised in Pakistan. LARC provide safe and effective protection against unintended pregnancies for an extended period of time with an added advantage of minimal user involvement. The dynamics of contraceptive usage in Pakistan point towards low usage of LARC, compared to short-term methods [1,2]. One of the possible reasons for low usage could be the fear of side effects [1,3,4].

The 2012–2013 Pakistan Demographic and Health Survey [1] reports a contraceptive prevalence rate of 35.4%, comprising 26.1% modern and 9.3% traditional method users. The most commonly reported methods used by married women of reproductive age (MWRA) include condoms (8.8%), followed by female sterilisation (8.7%), injectables (2.8%), IUDs (2.3%), pills (1.6%), and lactational amenorrhea (1.5%) [1]. Despite the evidence that long-term contraceptive methods, such as IUDs and implants, are more effective in reducing the total fertility rate (TFR) than short-term methods like condoms and pills [5], their use is less than desirable in Pakistan. On the other hand, female sterilisation, which is commonly used, is undertaken by women with an average age of 39 and only after 6+ children and thus may have limited impact on the TFR [6]. The greater reliance on short-term contraceptives in Pakistan lead to only a marginal decrease in the TFR from 4.1 in 2003–2006 to 3.8 births per woman in 2010–2012 [1,2]. The caveat with an increase in the contraceptive prevalence rate would be that, although desirable, it in itself is not the sole determinant for population reduction, which is in turn impacted by effective and persistent use of contraceptives. Changing the dynamics of contraceptive method mix with an increased share of LARC methods is therefore essential for achieving family planning (FP) goals in Pakistan.

High fertility and greater reliance on short-term contraceptive methods in Pakistan requires a rethinking of the FP strategy. Estimates suggest that only by switching 4% of current oral contraceptive users (about 100,000 MWRA) in Pakistan to IUDs or implants, more than 25,000 unintended pregnancies could be averted over a 5-year period [7]. If Pakistan is to achieve the FP 2020 commitments, it is imperative that the focus of FP interventions be reoriented towards enhancing IUD use (and other longer term methods such as implants) among young women in order to maximise the impact of IUD use on population stabilisation.

The Marie Stopes Society (MSS) Pakistan launched a quasi-experimental research project aimed at promoting healthy timing and spacing of pregnancies through increased use of IUDs in rural and under-served communities in eight districts in Punjab, Sindh, and Khyber Pakhtunkhwa (KP) provinces of Pakistan. These activities were aimed at supplementing the Government of Pakistan’s efforts targeting FP promotion and provision by providing a reliable evidence base with respect to effective FP intervention strategies that are relevant in the local context.

The 41-month (including 24 months of intervention) operations/operational research project tested two FP service delivery models: (1) the MSS social franchise model comprising a network of private FP and reproductive health service providers, branded as ‘*Suraj*’ (meaning Sun in English) [8]; and (2) a Community Midwife (CMW) provider intervention model comprising a Government of Pakistan cadre of skilled birth attendants [9]. The CMW intervention model is a partnership between MSS and the Maternal Newborn and Child Health programme of the Ministry of Health for the provision of quality contraceptive services in targeted communities through CMWs. The two models were selected for the larger quasi-experimental study in order to assess and compare the effectiveness and efficiency of each of the two intervention models with a control group based on an increase in contraceptive use.

Previous studies exploring LARC continuation rates in Pakistan have relied on retrospective data that are prone to recall bias [1,2,10]. Therefore, in addition to the pre- and post-cross-sectional surveys conducted for the larger study, we implemented a prospective client follow-up, only in the intervention arms, to examine IUD continuation rates. We focused on IUDs (instead of another type of LARC such as implants) because Pakistan’s national health policies do not allow the insertion of implants by a mid-level practitioner as it is considered a surgical procedure. Provision of LARC through the public sector CMW network was attempted for the first time in Pakistan through this project. Hence, prior to recommending a scaled up integration of LARC service provision with the CMW network at the national level, a study was warranted to test the effectiveness of the model in enabling continuous use of LARC by clients. The current study compared the effectiveness of the two interventions on IUD continuation at 24 months. Moreover, reasons of method discontinuation and its risk factors were studied in both of the intervention arms.

## Methods

### Study design and duration

The prospective client follow-up component was nested within the larger quasi-experimental research study. It was implemented in five districts in Punjab, Sindh, and KP provinces: the Suraj intervention model in districts Khanewal (Punjab), Nausheroferoze (Sindh), and Haripur (KP); and the CMW intervention model in districts Pakpattan (Punjab) and Rajanpur (Punjab). The Suraj model was implemented across three provinces of Punjab, Sindh, and KP while the CMW model was implemented in Punjab province only because: (1) CMWs were not operational in Sindh province then and (2) there were practical constraints of time, budgeting, and logistics in KP province. The recruitment of the study participants started in March 2011 and continued until September 2011. All participants were followed every second month for 24 months with the last follow-up conducted in November 2013.

### Selection of providers

A total of 50 service providers, 10 per district, comprising private providers in Suraj districts and CMW providers in CMW intervention districts were included in this study. All of the mid-level private providers were Lady Health Visitors with personal clinics because selection criteria for the Suraj network included (1) a proper clinic setup and (2) a practice in rural settings (at least 40 km away from District Health Quarter). Providers had a 2-year diploma in general healthcare provision and safe motherhood services. CMWs had no previous experience of delivering IUD services.

### Intervention description

Providers in both of the intervention models received basic clinical training on FP from MSS and were supported by male (1 for 10 service providers) and female community mobilisers (an outreach worker). The intervention components for the Suraj model are presented in Table 1. The CMW model differed from the Suraj intervention in two key aspects. First, the CMW intervention model did not include a voucher scheme for IUDs. Second, it did not include branding/marketing of CMW facilities in the community.

**Table 1:** **Description of intervention**

	**Intervention items**	**Description**
1	Training on reproductive health/family planning (FP) and post-training evaluation	Medical training: Reproductive health and FP, counselling, quality of services, and IUD insertion and removal Business training: Basic budgeting skills, record keeping, stock management, branding, marketing, and the voucher management The training was followed by a post-training evaluation conducted by an external consultant (medical doctor)
2	Female community mobiliser (FCM)	The FCM was a local resident of the community and underwent training on FP methods, the voucher distribution system, and record keeping; she also paid door to door visits, raised awareness, generated referrals, and distributed vouchers for the IUD to eligible women, identified through a poverty scale
	Each service provider was complemented with one FCM	
3	Male community mobilisers (MCM)	MCM was a local resident of the district; he underwent training and was responsible for targeting male community members; he formed community support groups comprised of key community stakeholders and conducted frequent *Mohallah* (neighbourhood) meetings
	There is one MCM per 10 service providers in a district	
4	Voucher for a long-term contraceptive method (IUD)	A voucher was worth PKRs 200 (US $2.27) and only for IUD (insertion, follow-up, and removal) services; a voucher could be redeemed at Suraj clinics; later the reimbursement was sent to the provider against her claim
5	Branding/marketing	Providers’ clinics were branded as ‘*Suraj*’ clinics while marketing was done through FCM, posters, wall paintings, leaflets, etc.; a signboard with a ‘*Suraj*’ logo was displayed above the gates of the Suraj clinics

### Enrolment of study participants

The first 50 women who were new acceptors of long-term or short-term contraceptive methods at each of the Suraj and CMW FP service facilities were asked to participate in the study. They were followed-up every 2 months for 24 months by respective MSS female community mobilisers in Suraj and CMW areas. Overall, 2,500 women were recruited for the study; however, the current analysis was performed amongst a subset (1,163) of the women who had received IUDs at the time of the recruitment. With a sample of 1,163 women (824 in Suraj and 339 in CMW), and assuming 22% cumulative probability of IUD discontinuation at 24-months [10], we could detect a difference of approximately 7 percentage points with 80% power.

Two types of clients were included in Suraj areas, (1) women paying out-of-pocket who had been referred to providers by Field Community Mobilisers for FP services, and (2) women who received a voucher for free IUD services after being assessed for wealth using a poverty ranking tool by the Field Community Mobilisers. The poverty ranking tool inquired about wealth status including the number of meals per day, household structure, number of household members, number of dependent members, source of fuel used for cooking, source of drinking water, sanitation, access to reproductive health services, and daily household income. Clients received a voucher if their score fell between the minimum score of 9 and 20 (inclusive) on a scale of 27.

### Data collection

During the follow-up visit, women were asked questions about current contraceptive use (including start date), method switching, method discontinuation (including stop date), reasons for discontinuation, method related complications, and pregnancy occurrence in case of method discontinuation. Data from participants who were lost at the follow-up stage were censored at the time of their last completed interview. Face-to-face interviews were conducted at participants’ homes in private except for the baseline interview, which was conducted at the healthcare facility.

The questionnaire was pre-tested in a similar setting and revised based on feedback. All female community mobilisers were trained on the questionnaire and were rigorously monitored during the course of data collection by the Principal (SKA) and Co-Investigators (WH, GM, and JA). Data entry operators were trained to observe the study protocol as well as data completeness, editing, and coding procedures. Data were entered using a specifically designed data entry programme using Visual FoxPro version 6.0. Local and central evaluation of collected data was carried out on a monthly basis.

### Data analysis

#### Study outcomes

The primary outcome of the study was IUD continuation rate. We defined IUD continuation as women who had an IUD inserted till the last follow-up while women were considered as IUD discontinuers if they had either switched to another contraceptive method (either another long-term, permanent, or short-term method) or had simply got an IUD removed or discontinued contraceptive use altogether.

#### Statistical analysis

The Statistical Package for Social Sciences software version 17.0™ was used for data analysis. We computed means, standard deviations, frequencies, and percentages as appropriate to describe the socio-demographic characteristics of women participating in the study. Differences in women’s characteristics and reasons for IUD discontinuation between intervention arms were assessed using χ^2^ and Fisher’s exact tests. We calculated the number of months of IUD use by capturing usage recorded between follow-up visits. Cumulative continuation probabilities were calculated for IUD usage using life-table analysis, a technique used to estimate survival probabilities (in this case, continuation probabilities) over time. Log rank and Breslow tests were run to determine differences, if any, in IUD continuation survival distributions between the intervention models and between the two types of clients.

Cox proportional-hazard analysis was used to determine risk factors associated with IUD discontinuation in terms of hazard ratios for overall as well as for each of the two intervention models separately. Risk factors with level of significance (or *P* value) less than <0.20 at the univariate level were considered for inclusion in the final model. The final multivariate model included adjustment for confounders that could potentially influence the outcome. The level of significance was set at a *P* value <0.05.

### Ethics statement

Verbal and written informed consent was obtained from the study participants. Data were stored on password protected computers, accessible only by authorised personnel. In order to ensure confidentiality, individual identifiers were removed at the time of analysis. The ethical approval for the project was provided by the Ethics Review Committee of the National Bioethics Committee of Pakistan (Ref no: 4-87/10/NBC-43/RDC/).

## Results

The distribution of study participants by districts and intervention models at baseline is presented in Table 2. A total of 824 women from the Suraj intervention and 339 women from the CMW intervention were included in the analysis. The loss to follow-up at the last visit was 2.4% (20/824) and 8.6% (29/339) in the Suraj and CMW intervention models, respectively. Study participants who were lost to follow-up were different from those who remained in the study in three aspects: they were more educated, had fewer living children, and the last child tended to be younger. In the Suraj model, 582 (70.6%) were voucher clients and 242 (29.4%) clients were paying out-of-pocket. In the CMW model, all clients were paying out-of-pocket.

**Table 2:** **IUD users by intervention model and district**

**District**	**Suraj (n = 824)**	**CMW (n = 339)**
Khanewal	272 (33.0%)	–
Haripur	282 (34.2%)	–
Naushahro Feroze	270 (32.8%)	–
Pakpattan	–	226 (66.7%)
Rajanpur	–	113 (33.3%)

### Sociodemographic characteristics

Table 3 summarises the sociodemographic and reproductive characteristics of participating women in the Suraj and CMW intervention models. Women in the Suraj intervention model were significantly younger (*P* <0.05), less illiterate (*P* <0.001), had a lower average monthly family income (*P* <0.05), and fewer living children than women in the CMW intervention model.

**Table 3:** **Socio-demographic characteristics of married women of reproductive age who had an IUD inserted between March and September 2011 by intervention model (Suraj vs. community midwives (CMW))**

**Indicators**	**Suraj intervention**	**CMW intervention**	***P*** **value**
	**n = 824 (%)**	**n = 339 (%)**	
Province			
Punjab	272 (33.0)	339 (100)	–
Sindh	270 (32.8)	–	–
Khyber Pakhtoonkhwah	282 (34.2)	–	–
Age of women, years			
15–25	232 (28.2)	71 (20.9)	<0.05
26–35	517 (62.7)	209 (61.7)	0.73
> 35	75 (9.1)	59 (17.4)	<0.05
Mean ± SD	28.9 (4.9)	31.0 (5.8)	<0.05
Women education level			
Illiterate	428 (51.9)	212 (62.5)	<0.001
Primary (1–5 years)	213 (25.8)	87 (25.7)	0.94
Secondary (6–10 years)	124 (15.0)	23 (6.8)	<0.001
Post-secondary	59 (7.2)	17 (5.0)	0.18
Monthly family income (PKR)			
≤ 3,000	106 (12.9)	51 (15.0)	0.32
3,001–6,000	331 (40.2)	130 (38.3)	0.56
6,001–9,000	212 (25.7)	67 (19.8)	<0.05
> 9,000	175 (21.2)	91 (26.8)	<0.05
Mean ± SD	7,493 (5,364)	8,375 (7,547)	<0.05
Number of living children^a^			
1–2	87 (14.5)	45 (16.1)	0.58
3–4	314 (52.2)	114 (40.7)	<0.001
5+	200 (33.3)	121 (36.4)	<0.01
Mean ± (SD)	4.2 (1.8)	4.5 (2.0)	<0.05
Age of last child, years			
Up to 1	286 (37.4)	177 (53.3)	<0.001
1–2	242 (31.6)	75 (22.6)	<0.05
> 2	237 (31.0)	80 (24.1)	0.07
Type of clients			
Voucher	582 (70.6)	––	–
Referral	242 (29.4)	339 (100)	–

^a^Missing Suraj intervention = 229, CMW intervention = 59.

### IUD continuation rates by Suraj and CMW interventions

Table 4 shows that the cumulative probability of IUD continuation at the first two intervals was higher amongst women in the CMW intervention model (98% and 94%) than women from Suraj areas (92% and 85%). However, at 24 months, the cumulative probability of IUD continuation was similar in both the intervention arms with Suraj at 82% and CMW at 80%. The probability distributions of IUD continuation were similar in both intervention models (Log rank test: χ^2^ = 0.06, *df =* 1, *P* = 0.81; Breslow test: χ^2^ = 0.6, *df =* 1, *P* = 0.44).

**Table 4:** **Cumulative probability of IUD continuation by intervention model (Suraj vs. community midwives (CMW))**

**Interval, months**	**Suraj intervention**			**CMW intervention**		
	**Women with IUD entering interval**	**IUD continuation (cumulative probability)**	**95% CI**	**Women with IUD entering interval**	**IUD continuation (cumulative probability)**	**95% CI**
0 to <6	824	0.92	(0.90–0.94)	339	0.98	(0.95–0.99)
6 to <12	753	0.85	(0.83–0.88)	331	0.94	(0.91–0.96)
12 to <18	681	0.83	(0.80–0.85)	314	0.89	(0.85–0.92)
18 to <24	655	0.82	(0.79–0.84)	292	0.80	(0.76–0.84)

### IUD continuation rates by type of client (paying out-of-pocket vs. voucher)

We compared the cumulative probability of IUD continuation between paying out-of-pocket and voucher clients. Table 5 shows that clients who paid out-of-pocket for IUDs had higher likelihood of discontinuation within first 6 months of its use than clients who had received IUD through vouchers (87% vs. 94%, respectively). However, no statistically significant differences were found between method discontinuation in subsequent intervals. At the end of the follow-up period, 84% of voucher clients and 77% of paying out-of-pocket clients were reported to be continuing IUD usage (Table 5). The probability distribution of IUD continuation was significantly greater in voucher clients compared to clients who paid for the services (Log rank test: χ^2^ = 5.23, *df =* 1, *P <*0.05; Breslow test: χ^2^ = 6.0, *df =* 1, *P <*0.05).

**Table 5:** **Cumulative probability of IUD continuation by type of clients (paying out-of-pocket vs. voucher)**

**Interval, months**	**Suraj intervention**					
	**Paying out-of-pocket**			**Voucher**		
	**Women with IUD entering interval**	**IUD continuation (cumulative probability)**	**95% CI**	**Women with IUD entering interval**	**IUD continuation (cumulative probability)**	**95 % CI**
0 to <6	242	0.87	(0.82–0.91)	582	0.94	(0.92–0.96)
6 to <12	207	0.81	(0.76–0.86)	546	0.87	(0.84–0.90)
12 to <18	182	0.78	(0.73–0.83)	499	0.85	(0.81–0.87)
18 to <24	175	0.77	(0.72–0.82)	480	0.84	(0.80–0.86)

### Method switching behaviour among women using IUDs

We recorded a total of 147 and 62 instances of IUD discontinuation in Suraj and CMW intervention models, respectively. The proportion of women discontinuing IUD usage without switching to any other method was not significantly different (*P* = 0.08) by intervention models and was 40.1% for Suraj and 53.2% for CMW intervention models, respectively (Table 6). Women switching to injections were 28 (19.0%) and 11 (17.7%) for Suraj and CMW interventions, respectively. Condoms were the most common method adopted by women in both intervention models after IUD discontinuation (Table 6).

**Table 6:** **Method-switching behaviour among women who discontinued IUD, by intervention model (Suraj vs. community midwives (CMW))**

**Contraceptive method after IUD discontinuation**	**Suraj intervention**	**CMW intervention**	***P*** **value**
	**n = 147 (%)**	**n = 62 (%)**	
Injections	28 (19.0)	11 (17.7)	0.83
Pills	11 (7.5)	2 (3.2)	0.25
Condoms	43 (29.3)	12 (19.4)	0.14
Permanent method/sterilisation	4 (2.7)	3 (4.8)	0.44
Abstinence	2 (1.4)	1 (1.6)	0.89
Discontinued contraceptive use	59 (40.1)	33 (53.2)	0.08

Table 7 describes the reasons for IUD discontinuation reported by women across Suraj and CMW interventions. Desire for more children (Suraj = 29.3%, CMW = 40.3%) and method-related side effects (Suraj = 57.1%, CMW = 38.7%) were reported as the most prominent reasons for IUD discontinuation amongst women in both intervention arms.

**Table 7:** Reasons for IUD removal by intervention model (Suraj vs. community midwives (CMW))

**Reasons for IUD removal**	**Suraj intervention**		**CMW intervention**		***P*** **value**
	**n = 147 (%)**		**n = 62 (%)**		
Want more children	43	(29.3)	25	(40.3)	0.12
Worry about side effects	10	(6.8)	8	(12.9)	0.15
Heavy bleeding	33	(22.4)	8	(12.9)	0.11
Irregular bleeding	14	(9.5)	2	(3.2)	0.12
Pain	16	(10.9)	1	(1.6)	<0.05
Infection	11	(7.5)	5	(8.1)	0.89
Husband disagreement	9	(6.1)	3	(4.8)	0.72
IUD self-dislodged	5	(3.4)	2	(3.2)	0.62
Husband died	2	(1.4)	1	(1.6)	0.89
Others^a^	4	(2.7)	7	(11.3)	0.09

^a^Did not want more children, sterilised, husband is abroad, spotting, weight gain, allergy, weakness, opposed by in laws, method not effective, method not available.

### Risk factors for IUD discontinuation

Tables 8 and 9 present risk factors for IUD discontinuation in Suraj and CMW intervention models, respectively. For women in the Suraj model, analysis shows that IUD discontinuation was significantly associated with geographical location or place of residence of women while adjusting for age, income, number of children/MWRA, type of client, and age of last child (Table 6). Women from KP province had a significantly lower hazard to discontinue (higher hazard to continue) IUD use compared to their counterparts from Sindh (HR = 0.26, 95% confidence interval (CI), 0.13–0.53; Table 8). A similar analysis of women in the CMW model demonstrated that younger women in the 15–25 years of age bracket had significantly greater risk of discontinuing IUD use compared to women older than 35 years of age, while adjusting for their education levels (HR = 2.44, 95% CI, 1.10–5.41; Table 9).

**Table 8:** **Unadjusted and adjusted hazard ratios for IUD discontinuation at 24 months, by sociodemographic and economic factors (Suraj intervention)**

**Characteristics**	**Suraj intervention**	
	**Unadjusted HR**	**Adjusted HR**
	**(95% CI)**	**(95% CI)**
Age of women, years		
15–25	8.32 (2.62–26.48)^a^	2.55 (0.73–8.94)
26–35	4.17 (1.32–13.19)^a^	1.97 (0.60–6.43)
> 35	1	
Province		
Sindh	1	
Punjab	1.44 (1.01–2.05)^a^	0.97 (0.59–1.60)
Khyber Pakhtunkhwa	0.33 (0.20–0.55)^a^	0.26 (0.13–0.53)^a^
Monthly family income (PKR)		
≤ 3,000	2.34 (1.26–4.33)^a^	0.79 (0.33–1.91)
3,001–6,000	2.17 (1.29–3.65)^a^	1.03 (0.54–1.95)
6,001–9,000	1.84 (1.05–3.23)^a^	1.12 (0.57–2.20)
> 9,000	1	
Number of living children		
1–2	1.55 (0.86–2.81)^b^	1.30 (0.63–2.66)
3–4	1.03 (0.64–1.65)^b^	1.03 (0.60–1.75)
5+	1	
Age of last child, years		
Up to 1	1.78 (1.15–2.76)^a^	1.13 (0.63–2.01)
1–2	1.52 (0.95–2.40)^b^	1.47 (0.83–2.61)
> 2	1	
Type of client		
Voucher	1	
Referral	1.48 (1.06–2.07)^a^	0.99 (0.61–1.59)

^a^Significant at *P* <0.05; ^b^Significant at *P* <0.20.

**Table 9:** **Unadjusted and adjusted hazard ratios for IUD discontinuation at 24 months, by sociodemographic and economic factors (community midwives (CMW) intervention)**

**Characteristics**	**CMW intervention**	
	**Unadjusted HR**	**Adjusted HR**
	**(95% CI)**	**(95% CI)**
Age of women, years		
15–25	2.18 (1.00–4.77)^b^	2.44 (1.10–5.41)^a^
26–35	1.01 (0.48–2.12)	1.11 (0.52–2.36)
> 35	1	
Education		
Illiterate	3.88 (0.53–28.15)^b^	4.36 (0.60–31.70)
Primary (1–5 years)	3.06 (0.41–23.19)	3.20 (0.42–24.29)
Secondary (6–10 years)	1.51 (0.14–16.63)	1.67(0.15–18.47)
Post-secondary	1	
Monthly family income (PKR)		
≤ 3,000	1.37 (0.67–2.79)	–
3,001–6,000	0.75 (0.40–1.41)	–
6,001–9,000	0.82 (0.39–1.75)	–
> 9,000		
Number of living children		
1–2	1.00 (0.42–2.40)	–
3–4	1.50 (0.81–2.76)	–
5+		
Age of last child, years		
Up to 1	0.91 (0.49–1.70)	–
1–2	1.06 (0.52–2.18)	–
> 2		

^a^Significant at *P* <0.05, ^b^Significant at *P* <0.20.

Table 10 presents adjusted hazard ratios for IUD discontinuation at 24 months estimated from combined datasets from both intervention sites. IUD discontinuation was associated with province: women living in KP province were less likely to discontinue use compared with women from Sindh province (Adjusted HR = 0.27, 95% CI, 0.13–0.54). Interestingly, no other potential factor showed a significant association with IUD discontinuation.

**Table 10:** **Adjusted hazard ratios for IUD discontinuation at 24 months, by sociodemographic and economic factors (for both intervention arms)**

**Characteristics**	**Adjusted HR (95% CI)**
Age of women, years	
15–25	1.88 (0.92–3.83)
26–35	1.23 (0.66–2.31)
> 35	1
Province	
Sindh	1
Punjab	1.07 (0.66–1.75)
Khyber Pakhtunkhwa	0.27 (0.13–0.54)^a^
Education	
Illiterate	1.88 (0.66–5.31)
Primary (1–5, years)	2.02 (0.71–5.8)
Secondary (6–10, years)	1.58 (0.51–4.87)
Post-secondary	1
Monthly family income (PKR)	
≤ 3,000	1.08 (0.59–1.97)
3,001–6,000	0.85 (0.52–1.38)
6,001–9,000	0.94 (0.55–1.59)
> 9,000	
Number of living children	
1–2	1.18 (0.67–2.07)
3–4	1.17 (0.78–1.77)
5+	1
Age of last child, years	
Up to 1	0.97 (0.62–1.52)
1–2	1.25 (0.78–2.00)
> 2	1
Type of client	
Voucher	1
Referral	1.07 (0.66–1.72)
Mode	
Suraj	1
Community midwives	0.74 (0.44–1.23)

^a^Significant at *P* < 0.05.

### Pregnancy occurrence

During the 24-month follow-up period, 111 women reported becoming pregnant. Among these, 83.8% (93/111) became pregnant after IUD discontinuation, while 16.2% (18/111) reported conceiving during IUD usage. The probability distribution of pregnancy among IUD continuing women was significantly lower compared to women who had discontinued IUD use (Log rank test: χ^2^ = 790.3, *df =* 1, *P <*0.01; Breslow test: χ^2^ = 725.1, *df =* 1, *P <*0.01).

## Discussion

The findings of this 24-month prospective client follow-up study indicate that both Suraj and CMW intervention models had very similar IUD continuation rates. At 12 months, the IUD continuation rate was higher in the CMW intervention (94%) than Suraj (85%). This trend changed at 24 months, when 82% and 80% of women in the Suraj and CMW models, respectively, reported IUD continuation. Although our data do not show one intervention to have a more significant impact than the other on IUD continuation rates, it is encouraging that both interventions were equally effective in maintaining higher IUD continuation rates compared to national data, which report the 12-month IUD continuation rate to be 75% [1].

Our findings are close to the earlier reported IUD continuation rate of 77.3% from a cross-sectional study in Pakistan [10]. The continuation rates in the intervention areas of this study are higher than those in other Asian countries [11,12]. High IUD continuation rates among women – served through the two intervention models – has important implications for the future of FP programmes, particularly with regard to the promotion and uptake of IUDs in the country and hence reduction of the fertility rate. It has been estimated that wider use of IUDs would reduce the overall number of unintended pregnancies more than any other modern contraceptive method [7].

Adjusted HRs showed no difference in the IUD discontinuation rates between vouchers and paying out-of-pocket clients, which is consistent with the previous study conducted in Pakistan [10]. This finding may indicate that the vouchers were probably distributed to women who had expressed a need for the FP method after proper assessment.

Moreover, the similarity of the IUD continuation rates between the Suraj and CMW models shows that strengthening health services supported with comprehensive capacity building training of mid-level providers on FP services, counselling, and ensuring reliable and smooth FP supplies can effectively enhance IUD continuation in Pakistan. Yet, we suggest further research should be conducted to understand how newly trained CMWs performed equally well on the provision of IUD compared with Lady Health Visitors who have more years of experience in this field.

The most common reasons for IUD discontinuation reported by the clients included desire to have more children and side-effects, such as heavy and irregular bleeding, pain, and infections, which are consistent with research previously conducted in Pakistan and elsewhere [10-12]. The desire for children and method-related side-effects were cited by a greater proportion of women in both the Suraj and CMW intervention models. We also found that IUD discontinuation was strongly linked with pregnancy occurrence and method switching. Since we did not record information on the intent of the pregnancy it would be difficult to ascertain whether the pregnancies were because of contraceptive failure (and hence IUD discontinuation and removal) or a reflection of a gap in contraceptive coverage while switching from IUDs to other methods. However, desire for children expressed by women as the major reason for IUD discontinuation may simply explain that the association is causal.

Substantial follow-up in both intervention models is the major strength of this study. In previous epidemiological cohorts, various authors have suggested follow-up rates of 50–80% as acceptable [13,14]. The study had few limitations. For example, there were differences in characteristics of women who dropped out of the cohort compared with women who had completed a 24-month follow-up. Moreover, the study did not have a control arm, which could have been compared with each of the intervention arms to isolate the true effect of the intervention. Finally, the analyses did not account for the differentials in provider characteristics between the two arms, where Suraj providers are established providers compared with CMW who were relative new cadre.

As this study was undertaken in rural areas of the eight districts of Pakistan, the findings should be generalised with caution. Further research to evaluate the IUD continuation rates with a broader and diversified base of women can provide viable research evidence on the factors that determine IUD continuation and the cost effectiveness of each intervention in order to promote inexpensive intervention models. Finally, qualitative research should be conducted to understand why women in KP were less likely to discontinue than women in Sindh.

## Conclusion

The IUD continuation rate at 24 months was not significantly different between CMW and Suraj intervention models. The reasons for method discontinuation, including desire for more children and side effects, were found to be similar across the intervention arms and largely in line with published literature. The study findings demonstrate that trained mid-level private providers and outreach workers, supported with vouchers for free IUD services, in social franchising programmes can effectively promote IUD continuation. The findings also reveal that CMWs and Lady Health Visitors are equally capable of providing quality IUD services and ensuring higher method continuation. In light of these study findings, the government Maternal Newborn and Child Health programme could consider training CMWs on IUDs implementation and provision.

## Abbreviations

CMW: Community midwife
FP: Family planning
IUD: Intrauterine device
KP: Khyber Pakhtunkhwa
LARC: Long-acting reversible contraceptives
MSS: Marie Stopes Society
MWRA: Married women of reproductive age
TFR: Total fertility rate
FCM: Female Community Mobiliser
